# Exotic Fish in Exotic Plantations: A Multi-Scale Approach to Understand Amphibian Occurrence in the Mediterranean Region

**DOI:** 10.1371/journal.pone.0129891

**Published:** 2015-06-10

**Authors:** Joana Cruz, Pedro Sarmento, Miguel A. Carretero, Piran C. L. White

**Affiliations:** 1 Environment Department, University of York, Heslington, York, YO10 5DD, United Kingdom; 2 CIBIO Research Centre in Biodiversity and Genetic Resources, InBIO, Universidade do Porto, Campus Agrário de Vairão, Rua Padre Armando Quintas, 7. 4485–661 Vairão, Vila do Conde, Portugal; 3 CESAM, Universidade de Aveiro, Campus Universitário de Santiago, 3810–193, Aveiro, Portugal; Universität Zurich, SWITZERLAND

## Abstract

Globally, amphibian populations are threatened by a diverse range of factors including habitat destruction and alteration. Forestry practices have been linked with low diversity and abundance of amphibians. The effect of exotic *Eucalyptus* spp. plantations on amphibian communities has been studied in a number of biodiversity hotspots, but little is known of its impact in the Mediterranean region. Here, we identify the environmental factors influencing the presence of six species of amphibians (the Caudata *Pleurodeles waltl*, *Salamandra salamandra*, *Lissotriton boscai*, *Triturus marmoratus* and the anurans *Pelobates cultripes* and *Hyla arborea/meridionalis*) occupying 88 ponds. The study was conducted in a Mediterranean landscape dominated by eucalypt plantations alternated with traditional use (agricultural, *montados* and native forest) at three different scales: local (pond), intermediate (400 metres radius buffer) and broad (1000 metres radius buffer). Using the Akaike Information Criterion for small samples (AIC_c_), we selected the top-ranked models for estimating the probability of occurrence of each species at each spatial scale separately and across all three spatial scales, using a combination of covariates from the different magnitudes. Models with a combination of covariates at the different spatial scales had a stronger support than those at individual scales. The presence of predatory fish in a pond had a strong effect on Caudata presence. Permanent ponds were selected by *Hyla arborea/meridionalis* over temporary ponds. Species occurrence was not increased by a higher density of streams, but the density of ponds impacted negatively on *Lissotriton boscai*. The proximity of ponds occupied by their conspecifics had a positive effect on the occurrence of *Lissotriton boscai* and *Pleurodeles waltl*. Eucalypt plantations had a negative effect on the occurrence of the newt *Lissotriton boscai* and anurans *Hyla arborea/meridionalis*, but had a positive effect on the presence of *Salamandra salamandra*, while no effect on any of the other species was detected. In conclusion, eucalypts had limited effects on the amphibian community at the intermediate and broad scales, but predatory fish had a major impact when considering all the scales combined. The over-riding importance of introduced fish as a negative impact suggests that forest managers should prevent new fish introductions and eradicate fish from already-occupied ponds whenever possible.

## Introduction

Amphibians are one of the most threatened vertebrate groups, with nearly one third of the total number of species now at risk of extinction [1]. In the Mediterranean region, one of the global biodiversity hotspots [2], 29% of amphibian species are threatened with extinction, with habitat alteration and fragmentation cited as the primary reasons for past and future extinctions [1, 3]. One change in land use that has contributed to these problems in the Mediterranean has been the spread of forest plantations, which expanded by 5 million ha per year between 2000 and 2010 [4]. Forestry practices have been associated with low diversity and abundance of amphibians, due to land cover disturbance, alteration of microclimates, and exposure during terrestrial phase, mainly affecting forest specialist species [5–9]. Practices such as clear-cutting may lead to abrupt increase in surface temperature and loss of soil-litter moisture [10], decreasing survival and causing poor body condition [11], as well as altering migration behaviour [12] and connectivity. These processes may ultimately lead to species extirpation in the affected area [13, 14].

Exotic trees are used commonly in preference to native ones for forestry worldwide [15]. Eucalypt is one of the most commonly-planted trees in the world [4], and the negative impacts of forestry can expect to be exacerbated in exotic plantations [16]. Eucalypt has been associated with altered soil conditions, leading to lower pH in both soil [17] and water, disturbing the aquatic macroinvertebrate community, which is a key food source for amphibians [18], and also causing water depletion [19]. Changes in the soil and land characteristics may have a negative impact during aestivating and overwinter periods, especially on fossorial species. The effects of eucalypt plantations on amphibian communities have been documented in some biodiversity hotspots outside the eucalypt native range. In Madagascar [20], Brazil [21] and South Africa [16], where species richness is lower when compared to native forests; Costa Rica, where eucalypt plantations were a suitable habitat for the *Eleutherodactylus coqui* [22]; and USA, where the species richness was similar to native forests although differed in composition [23]. However, little equivalent research has been carried out in the Mediterranean basin hotspot [24], despite the widespread occurrence of eucalypt plantations in the region and the possible impact on habitat connectivity.

Connectivity is crucial for amphibians due to: (1) their distinct habitat requirements for feeding, breeding and overwintering; (2) an obligatory aquatic reproductive phase, for the Mediterranean species; (3) seasonal terrestrial adult migrations which make them susceptible to changes in landscape structure; (4) juvenile dispersal; and (5) their low vagilities and high risk of desiccation [3, 25]. Most estimates of migration and dispersal distance for the Mediterranean species (or related species) do not exceed 400 and 1000 metres, respectively [26–29]. Nevertheless, there are accounts of individuals exceeding this distance [29]. Land cover, the network of ponds and streams (ephemeral, temporary and permanent), and the proximity of other ponds occupied by their conspecifics are covariates that can influence connectivity of amphibian populations [30, 31]. The proximity and high density of ponds and streams may provide a route for migration, facilitating the movements whilst maintaining moist conditions [25]. During migration or dispersal, the preference for occupied ponds by conspecifics is common [30]. However, migration and dispersal are species- and individual-specific and influenced by the ability to overcome predation, challenging microclimatic conditions and the resistance to movements of the substrates [32–34]. For instance, clearcut areas may be more difficult to cross due to exposure to more extreme weather conditions and lack of refuge than habitats with vegetation cover [35]. Nevertheless, landscape processes are not the onlyones to affect amphibians’ population dynamics. At the local scale, pond characteristics also restrict occupation by certain species. The absence of exotic fish [36–38], presence of temporary ponds [39] and the presence of aquatic vegetation [40, 41] may all favour a diverse amphibian community. Due to their different life stages and requirements (embryo, larvae, juvenile and adult), amphibians make use of resources at various scales, so a cross-scale analysis can be valuable to assess the presence of a certain species.

There has been previous research worldwide to investigate local- and landscape-scale variables influencing occurrence patterns in amphibians, e.g. [42, 43], but the results are highly variable [44, 45] and region- and context-specific [42, 46]. Given the significance of the Mediterranean region for native biodiversity [47], including amphibians, and the predominance of eucalypt forest cover in the region, there is an urgent need to evaluate the impact of these plantations on the amphibian community and assess local and landscape-scale covariates of species occurrence.

Here, we evaluate the impacts of different landscape and environmental factors on six species of amphibians (the Caudata *Pleurodeles waltl*, *Salamandra salamandra*, *Lissotriton boscai* and *Triturus marmoratus* and the anurans *Pelobates cultripes* and *Hyla arborea/meridionalis*). We evaluated the factors affecting pond occupancy by these species at three different scales, appropriate to the scale of individual ponds (local), migration distances (intermediate; 400 m) and dispersal distances (broad; 1000 m). Using different scales is useful as different variables may only become significant at a specific scale, improving the quality of the models [42, 46, 48, 49].

At local scale, we tested the hypothesis that all amphibian species would survive in ponds without fish, with a temporary hydroperiod and with high aquatic vegetation cover. At the intermediate and broad scales, we hypothesised that amphibian species occurrence in a pond would increase with the density of streams and ponds, and decline with increasing eucalypt cover and distance to streams. Finally, we hypothesised that amphibian species occurrence would be explained better by a combination of covariates across each scale than by covariates at any one scale.

## Materials and Methods

### Study Area

More than one-third of mainland Portugal is covered by forest (35%). Within this forested area, eucalypt (*Eucalyptus* spp.) is the dominant tree (26%), and both Maritime pine (*Pinus pinaster*) and native cork oak (*Quercus suber*) occupy 23% each [50]. We carried out the study in central-east Portugal, Castelo Branco district (39°40’– 40°10’N, 7°0’– 7°35’W). The area has a Mediterranean climate, with a mean temperature of 16.7°C (mean minimum: 11.0°C; mean maximum: 22.4°C) and an average precipitation of 758 mm [51].

**Fig 1:**
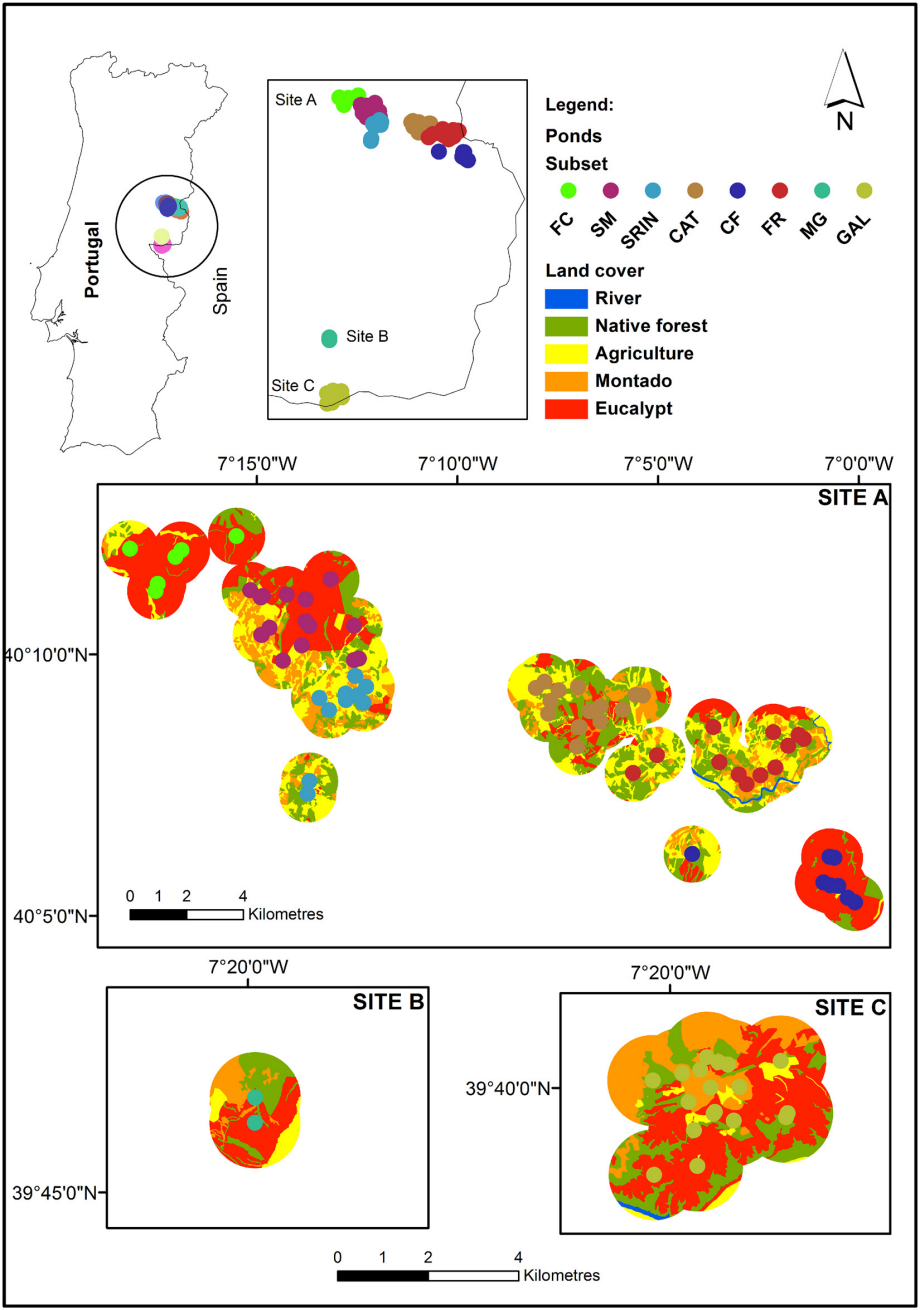
The location of the study area. Distribution of the 88 ponds surveyed monthly between February and June 2011 in central-east Portugal. There were three major study sites distributed in the region: Site A, with 67 ponds; Site B: 2 ponds; Site C: 19 ponds. Each site was divided into subsets (FC, SM, SRIN, CAT, CF. FR, MG, GAL), according to geographical, topographical or barrier features.

In the study area, the forest land cover is dominated by eucalypt (*Eucalyptus globulus*) plantations (36%), with different age stands, natural forest of cork oak (*Quercus suber*) and Holm oak (*Quercus ilex*), Maritime pine plantations, scrubland areas dominated by *Cytisus* spp., *Cistus* spp. and *Erica* spp. (all comprising 23%), and *montados* (oak savannah-like woodland) (16%). In addition to forestry, the landscape is used patchily for livestock grazing, olive (*Olea europaea*) groves, wheat (*Triticum* spp.) production, and small-scale subsistence agriculture (24%) (Fig 1). Most of the eucalypt stands are on their third rotation, planted for the first time in the mid-1970s. Each rotation lasts between 12 to 16 years depending on site productivity and plantations are managed by coppicing. The *montados* in the study area are actively exploited, with cattle grazing and cork extraction.

### Field sampling

We selected the ponds in order to represent roughly the proportion of habitats in the landscape. We also wanted to have spatial replicates of the ponds in the different habitats, that is why we chose ponds in eucalypt plantations that geographically were similar and near agriculture land and montados. We tried to sample all the ponds that were in a particular area as long as we had permission from the Estate managers. We recorded the presence/absence of six species—the urodeles Iberian ribbed newt (*Pleurodeles waltl*) (PW), fire salamander (*Salamandra salamandra*) (SS), Bosca's newt (*Lissotriton boscai*) (LB) and marbled newt (*Triturus marmoratus*) (TM), and the anurans Western spadefoot (*Pelobates cultripes*) (PC) and tree frog (*Hyla arborea/meridionalis*) (HY). Sampling was carried out monthly from February to June 2011, in 88 ponds, distributed in three major areas (sites A, B and C; Fig 1). Considering the different detectability of the species studied and their life stages, we used a range of techniques to increase the detectability and to record their presence/absence: dipnetting, visual surveys and acoustic night surveys. [52] refer that to achieve 95% of detection probability for all amphibian species in the Mediterranean region, a minimum of three visits is required, spread out during the breeding season and including a combination of nighttime call count, nighttime visual encounter and daytime netting. We surveyed the ponds five times, and applied all the methods each time we did so. If one species was detected by one single method once, it was considered to be present in that pond. During each visit, surveys were conducted by two independent observers. Each observer began their surveys at opposite sides of the water body and walked around the perimeter of the pond in the opposite direction, separately recording detections of all life stages of encountered amphibian species. Sampling effort was proportional to the pond size. Dipnetting was complemented with visual surveys in and around each water point to detect eggs, larvae, juveniles and adults. Amphibians were identified to the species level, whenever possible, using identification keys [53, 54]. Tree frog *Hyla arborea/meridionalis* tadpoles were identified to genus, because they could not be reliably identified in the field [55].

The visual surveys took place during day and night-time, the latter with the aid of torchlight (Streamlight, model Fire Vulcan Led). For the night survey, we had a 1-minute pause after arrival and then conducted a 3-minute aural survey, in which we identified each species call, before the visual survey started [26].

Amphibian data were collected following all legal requirements and we had the needed permits by Instituto da Conservação da Natureza e da Biodiversidade (ICNB) (the national authority for nature conservation and wildlife protection) to sample protected species. The ICNB permit was issued under the EU Habitats Directive and considered safety measures to avoid spread of any pathology, namely the disinfection of equipment with 1% hypochlorite solution. Considering this safety measure and the fact that no animals were sacrificed there was no need for approval by any animal ethics committee. The sampling within private land was performed with the authorization of the land owners.

The authors confirm that all data underlying the findings are fully available without restriction. All data files are available from the corresponding author’s Research Gate website upon request.

### Local scale

At each pond, we recorded the presence of predatory fish (FISH), the hydroperiod (HYDRO), soil type (muddy or shale) (SOIL) and the percentage of aquatic vegetation [floating (FLOAT), emergent (EMER) and submerged (SUBMER)]. The presence of predatory fish was assessed while doing the surveys by visual observation and interviewing the estate managers. We recorded the presence of pumpkinseed sunfish (*Lepomis gibbosus*), eastern mosquito fish (*Gambusia holbrooki*) and largemouth bass (*Micropterus salmoides*); these species are non-native to the region and classified as invasive under national law. We divided the hydroperiod into two levels: temporary (retains water between 3 to 6 months) and permanent (maintains water all year around), based on previous knowledge by the surveyors and, when necessary, confirmed by the estate managers. The soil type was assessed by visual observation, and it was classified as muddy or shale depending if it was an earthy or rock substrate, respectively.

The aquatic vegetation estimated visually by the same surveyor and classified into three classes: floating—plants rooted or free that float on the water surface, like *Ranunculus* spp.; emergent—rooted plants that grow above the water surface, such as *Typha* spp.; submerged—rooted plants that grow up to the water surface but not above it.

### Intermediate scale

The variables at this scale were measured within a 400 m radius buffer of each pond, using ESRI ArcGIS 10 and the land cover map of Portugal, from 2007 (COS2007) [56] and the Ordnance Survey maps.

The proportion of each land cover level was classified according to the main classes [agriculture (AGRIC), eucalypt plantations (EUC), *montados* (MONT) and native forest (NATFOR)]. We applied the data exploration guidelines described by [57] to the datasets for each of the six individual species to assess collinearity between the predictors and possible interactions. We carried out interaction plots and according to their results (non-consistent effect across all levels) we chose those interactions. Hence, we added to the models the interaction AGRIC400:MONT400 to the models. The distance to the nearest streams was estimated for both ephemeral streams (hydrologically dependent on rainfall, and are dry for most of the year, retaining water for less than 4 weeks after the last rainfall event) (NEPH) and temporary streams (retaining water for more than 6 months in a year) (NTEMP). Distance to ponds (NPOND) was also estimated. Following initial data exploration, we added the interaction term NEPH:NPOND. The density of ephemeral and temporary streams (DEPH and DTEMP) and ponds (DPOND) was also calculated. For each amphibian species we also measured the distance to the nearest other pond occupied by their conspecifics during the current survey (NPW, NSS, NLB, NTM, NPC, NHY).

### Broad scale

To investigate habitat associations at a broad scale, we assessed the same variables as in the intermediate scale—land cover, the distance to the nearest ephemeral and temporary streams, distance to ponds and density of ephemeral and temporary streams and ponds, and the distance to the nearest other pond occupied by their conspecifics—but we applied a 1000 m buffer around each pond. Following data exploration and analysis of the interaction plots, we added the interaction AGRIC1000:NATFOR1000.

### Model building and model selection

As mentioned before, we followed the data exploration guidelines of [57]. To assess collinearity, we used the Spearman rank correlation coefficient |r| because it makes no assumption about function of the relationships between two variables [58]; |r| > 0.6 was chosen to indicate high collinearity between variables, and where this was found, the variables were not used together in the same model. This value was chosen as a compromise, since the threshold for high collinearity is defined by some authors as |r| > 0.5 [58], whereas other authors propose a value of |r| > 0.7 [59].

**Fig 2:**
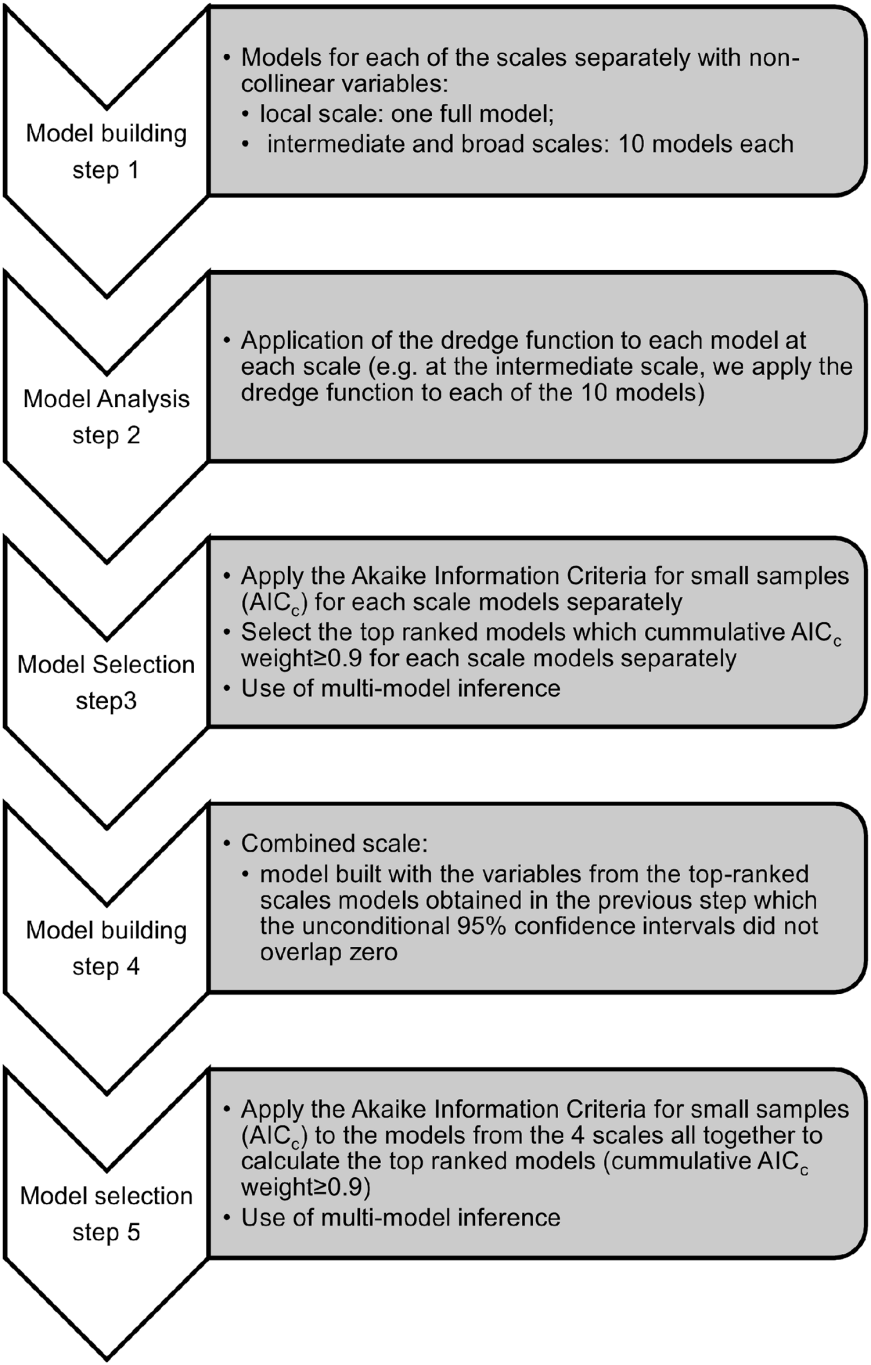
Flowchart describing the statistical analysis applied to the data.

In order to determine whether there was a difference in the studied response variables between eucalypt plantation and other land covers (agriculture, *montados* and native forest), we applied a chi-square test for multiple independent samples followed by post-hoc pairwise comparisons using False Discovery Rate method (R Package “fifer”) [60]. To assess which predictors better explained the behaviour of the response variables, we used a generalised linear mixed model (GLMM) fit to Laplace approximation, with a binomial error distribution (to model species occurrence) with the local subsets as the random variables, using package glmmADMB [61]. The subsets were defined according to geographical, topographical or barrier features (e.g. roads) which created eight local subsets (Fig 1), to account for spatial intercorrelation. The steps taken to determine the best top-ranked models are explained in Fig 2. Firstly, we combined all covariates, avoiding multicollinearity, in (1) one single full-model at a local scale, (2) 10 models at an intermediate scale, and (3) 10 models at a broad scale (Table A in S1 File). At the local scale the variables were not collinear unlike at the intermediate and broad scale and that is the reason for the different number of models. There was collinearity between agriculture cover and eucalypt cover at both scales (intermediate r_S_ = -0.79, p < 0.001; broad r_S_ = -0.79, p < 0.001), and between eucalypt cover and montados (intermediate r_s_ = -0.63, p < 0.001; broader scale r_S_ = -0.64, p < 0.001). Secondly, we used data dredge statistics (dredge—MuMIn R package) [62] to run GLMM on those models. Dredge statistics are a valid method, used when multiple variables may contribute to a response behaviour and it is important not to exclude any combinations of these variables. All the variables that we used have been described as significant determinants of amphibian occurrence in the literature. We did not want to omit any of these potentially important variables, especially since the effect of eucalypt stands on amphibians is poorly studied in the Mediterranean area. In addition, when considering a guild of species, it is difficult to choose an assemblage of variables that is relevant for all the species. In such cases, dredging is a recommended procedure. Recent papers covering genetics [63, 64], carnivore monitoring [65], amphibian studies [66] and bird movements and fragmentation [67] have all used dredge statistics.

Thirdly, we approximated model parsimony, given our data and model set by species, using Akaike Information Criterion for small samples (AIC_c_) [68] and weighted the support of each model using AIC_c_ weights [69]. We used model averaging to determine the direction and magnitude of the effect of each predictor variable and we report significantly positive or negative effects when 95% confidence intervals of parameter estimates did not contain zero [69–72]. The variables chosen to build the combined scale models were selected using the results from the previous steps, i.e., variables from the models which Akaike weights was more or equal to 0.9 and which confidence intervals did not overlap zero, in case of multiple models, of each scale (local, intermediate and broad). The number of full models varied according to the species (Table A), and model selection was applied using their ΔAIC_c_ and Akaike weights. Finally, we considered all together the top ranked models from the four scales and ranked them according to their ΔAIC_c_ and Akaike weights and averaged the models coefficients which cumulative Akaike weights was ≥0.9. The relative importance of each predictor variable [69] was then calculated based on AIC_c_ weights (‘importance’ function in ‘MuMIn’).

In order to evaluate the effect size of each covariate, we calculated the odds ratio (OR) using the model average parameter estimates for each response variable [73]. Odds ratios >1 indicate a positive effect; ratios <1 indicate a negative effect. Only variables for which the confidence interval of the coefficient did not overlap zero and cumulatively had an OR≥1.1 or OR≤0.90 were plotted.

## Results

All of the studied species were detected at least once in each dominant land cover (Table 1). The most common species were *Hyla arborea/meridionalis*, *L*. *boscai*, *P*. *waltl* and *T*. *marmoratus*, all of which were present in more than 60% of the surveyed ponds.

**Table 1:** Species occurrence.

	AGRIC	EUC	MONT	NATFOR	AGRIC	EUC	MONT	NATFOR	Total
	Intermediate				Broad				
*Pleurodeles waltl*	21 (0.81±0.08)	18 (0.50±0.08)	11 (0.61±0.12)	7 (0.88±0.12)	28 (0.90±0.05)	19 (0.49±0.08)	7 (0.64±0.15)	3 (0.43±0.20)	57 (0.65±0.05)
*Salamandra salamandra*	8 (0.31±0.09)	26 (0.72±0.08)	2 (0.11±0.08)	3 (0.38±0.18)	9 (0.29±0.08)	27 (0.69±0.07)	2 (0.18±0.12)	1 (0.14±0.14)	39 (0.44±0.05)
*Lissotriton boscai*	15 (0.58±0.10)	28 (0.78±0.07)	13 (0.72±0.11)	4 (0.50±0.19)	18 (0.58±0.09)	30 (0.77±0.07)	8 (0.73±0.14)	4 (0.57±0.20)	60 (0.68±0.05)
*Triturus marmoratus*	17 (0.65±0.10)	22 (0.61±0.08)	2 (0.67±0.11)	6 (0.75±0.16)	23 (0.74±0.08)	22 (0.56±0.08)	8 (0.73±0.14)	4 (0.57±0.20)	57 (0.65±0.05)
*Pelobates cultripes*	18 (0.69±0.09)	9 (0.25±0.07)	9 (0.50±0.12)	4 (0.50±0.19)	23 (0.74±0.08)	10 (0.26±0.07)	5 (0.45±0.16)	2 (0.29±0.18)	40 (0.45±0.05)
*Hyla arborea/meridionalis*	25 (0.96±0.04)	27 (0.75±0.07)	16 (0.89±0.08)	6 (0.75±0.16)	30 (0.97±0.03)	29 (0.74±0.07)	10 (0.91±0.09)	5 (0.71±0.18)	74 (0.84±0.04)
Number of ponds	26	36	18	8	31	39	11	7	88

Presence and proportion and standard error (between brackets) of each species according to the dominant land cover at each spatial scale (intermediate and broad) of the 88 ponds surveyed and total number of ponds where the species was found. AGRIC—agricultural; EUC—eucalypt plantations; MONT—*montados*; NATFOR—native forests

### Pleurodeles waltl

**Fig 3:**
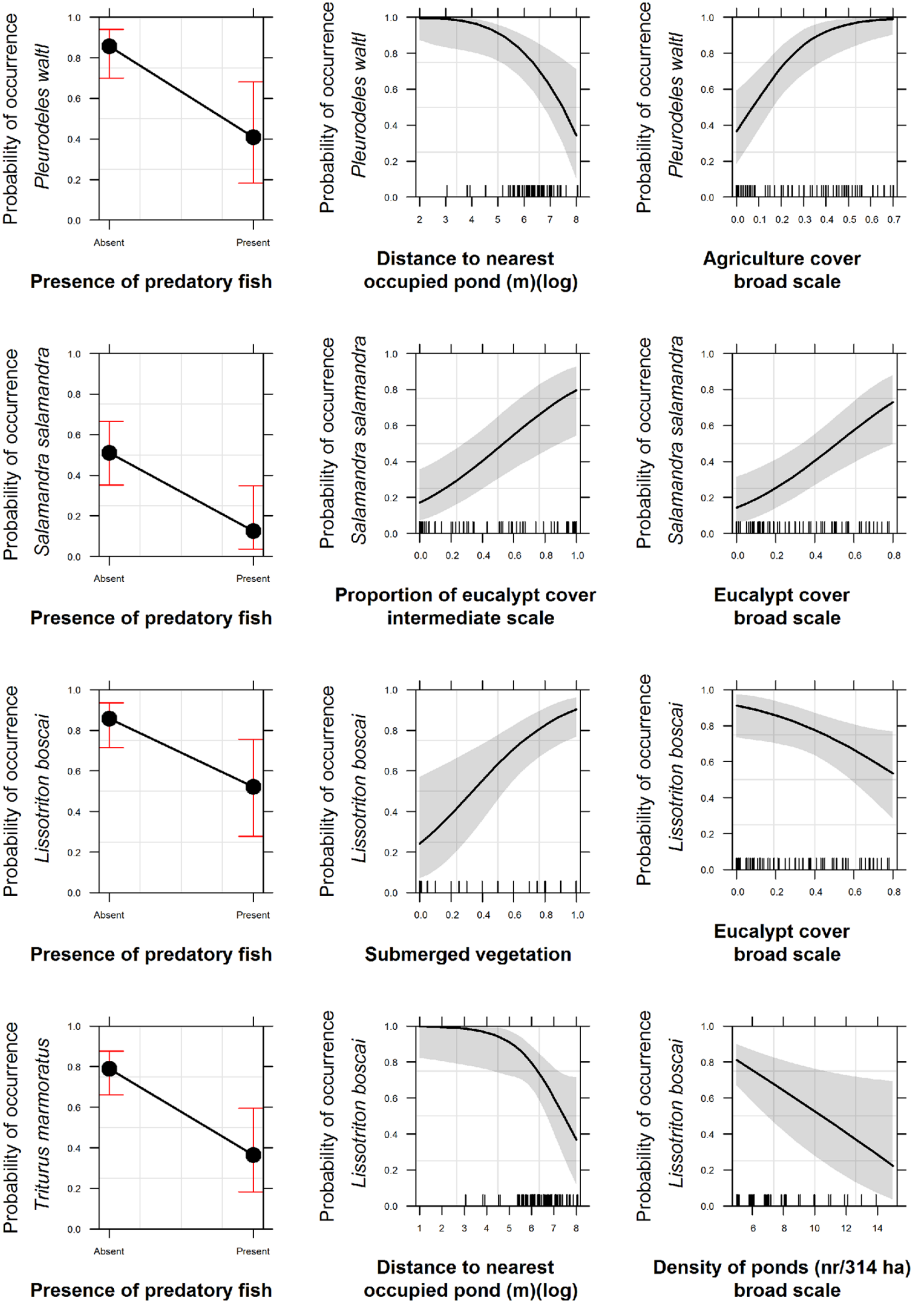
Fitted values predicted by the top ranked model for the Caudata for each of the response variables with strong effect according to the odds ratio results. The dashed line are the confidence intervals at 95%.

*P*. *waltl* presence was significantly different between eucalypt and native forest and agriculture and native forests at the intermediate scale (χ^2^ = 10.28, df = 3, P<0.05) and the broad scale ((χ^2^ = 32.72, df = 3, P<0.001) (Table 1;Table B in S1 File). At broad scale only, *P*. *waltl* presence was significantly different between agriculture and *montados*, and between eucalypt stands and *montados* (Table B in S1 File) *P*. *waltl* presence was negatively associated strongly with the presence of predatory fish (OR = 0.12, IC_95%_ [0.03–0.55]) and distance to the nearest other pond occupied by conspecifics (OR = 0.36, IC_95%_ [0.16–0.82]) (Table 2 and Fig 3). The model selection results provided strong support for a positive relationship between the probability of presence of *P*. *waltl* and the proportion of agricultural land at the broad scale (OR = 1393.29, IC_95%_ [16.59–1.17e^05^]). The remaining variables whose confidence intervals did not overlap zero—distance to the nearest ephemeral stream, distance to the nearest pond, and their interaction—did not influence substantially the occurrence of *P*. *waltl* (OR≈1) (Table 2).

**Table 2:** Model averaged parameter estimates (β) (top-ranked models) for each of the Caudata, odds ratio (OR) and respective 95% confidence intervals (IC_95%_).

Covariates	Pleurodeles waltl			Salamandra salamandra			Lissotriton boscaii			Triturus marmoratus		
	β (importance)	OR	IC_95%_	β (importance)	OR	IC_95%_	β (importance)	OR	IC_95%_	β (importance)	OR	IC_95%_
FISH	**-2.13 (1)**	0.12	0.03–0.55	**-1.98 (1)**	0.14	0.03–0.68	**-1.71 (1)**	0.18	0.05–0.70	**-1.93 (1)**	0.15	0.05–0.47
EMER										0.92 (0.24)	2.50	0.49–12.81
FLOAT										1.29 (1)	3.63	0.93–14.23
SUBMER							**3.37 (1)**	29.10	3.78–223.90	0.81 (0.19)	2.24	0.36–13.90
NPW	**-1.03 (1)**	0.36	0.16–0.82									
NLB							**-0.95 (1)**	0.39	0.17–0.89			
NTM										-0.58 (0.84)	0.56	0.29–1.07
NEPH	**4.39e** ^**-02**^ **(1)**	1.04	1.00–1.09	**-0.03 (1)**	0.97	0.94–0.99						
NTEMP	-7.98e^-04^ (0.37)	1.00	1.00–1.00									
NPOND	**3.24e** ^**-03**^ **(1)**	1.00	1.00–1.01									
NEPH:NPOND	**-1.08e** ^**-04**^ **(1)**	1.00	1.00–1.00									
EUC400				**2.94 (0.32)**	18.85	3.39–104.65						
AGRIC1000	**7.24 (1)**	1393.29	16.59–1.17e^05^									
NATFOR1000				-3.18 (0.29)	0.04	0.0004–4.10						
EUC1000				**3.63 (0.68)**	37.56	4.66-302-76	**-2.76 (1)**	0.06	0.005–0.75			
DTEMP1000							**-0.001 (1)**	1.00	1.00–1.00			
DPOND1000							**-0.27 (1)**	0.76	0.61–0.96			

Covariate importance between brackets. In bold are the covariates which confidence intervals do not overlap zero. Acronyms are explained in the text.

The two top ranked models combined local and broad scale variables and accounted for 0.95 of the model’s Akaike weights (Table C in S1 File). Models at the different scales considered individually had little support (ΔAIC_c_>6: ΔAIC_c_ Broad scale<ΔAIC_c_ Local scale<<ΔAIC_c_ Intermediate scale) (Table C in S1 File). The intermediate scale models had the weakest support, with the confidence intervals of the covariates density of ephemeral streams, density of temporary streams and proportion of agricultural land all overlapping zero (Table C in S1 File).

### Salamandra salamandra

*S*. *salamandra* presence was significantly different between eucalypt stands and agriculture, between eucalypt stands and *montados*, and between eucalypt stands and native forest at the intermediate (χ^2^ = 42.99, df = 3, P<0.001) and broad scales (χ^2^ = 50.15, df = 3, P<0.001) (Table 1; Table B in S1 File). At the broad scale only, there was also significant difference regarding *S*. *salamandra* occurrence between agriculture and native forests (Table B in S1 File). The model averaged parameter estimates provided strong support for a negative influence of fish (OR = 0.14, IC_95%_ [0.03–0.68]) and a positive influence of the proportion of eucalypt on *S*. *salamandra* presence—intermediate (OR = 18.85, IC_95%_ [3.39–104.65]) and broad scale (OR = 37.56, IC_95%_ [4.66–302.76]) (Table 2 and Fig 3). The distance to ephemeral streams did not have a strong impact on *S*. *salamandra* occurrence (OR = 0.97, IC_95%_ [0.94–0.99]) (Table 2 and Fig 3). The three top-ranked models accounted for 0.94 of the model’s Akaike weights and incorporated local, intermediate and broad scale covariates together (Table C in S1 File). When considered separately, models at each scale had little support, with ΔAIC_c_>5.5 (ΔAIC_c_ Intermediate scale ≈ ΔAIC_c_ Broad scale << ΔAIC_c_ Local scale) (Table C in S1 File). Some of the covariates measured across the three scales had little support, overlapping zero in their confidence intervals (proportion of native forest at broad scale, distance to pond and to temporary streams, at both temporary and intermediate scale, and hydroperiod and submerged vegetation, at the local scale) (Table C in S1 File).

### Lissotriton boscai

*L*. *boscai* presence was significantly different between agriculture and native forest, eucalypt stands and *montados*, and eucalypt stands and native forest and *montados* and native forest, at intermediate (χ^2^ = 23.63, df = 3, P<0.001) and broad scales (χ^2^ = 32.47, df = 3, P<0.001); and between agriculture and eucalypt stands, at the intermediate scale alone (Table 1; Table B in S1 File). A single top-ranked model with a combination of covariates from the local and broad scales best explained *L*. *boscai* presence, accounting for 0.99 of the model’s weight selection (Table C in S1 File). The single scale models had a ΔAIC_c_>10 (ΔAIC_c_ Intermediate scale ≈ ΔAIC_c_ Broad scale << ΔAIC_c_ Local scale). There was strong evidence of a negative relationship between occurrence of *L*. *boscai* and the presence of fish (OR = 0.18, IC_95%_ [0.05–0.70]), distance to the nearest other pond occupied by their conspecifics (OR = 0.39, IC_95%_ [0.17–0.89]), density of ponds at the broad scale (OR = 0.76, IC_95%_ [0.61–0.96]) and proportion of eucalypt at the broad scale (OR = 0.06, IC_95%_ [0.005–0.75]) (Table 2 and Fig 3). The proportion of submerged aquatic vegetation had a positive and strong influence on *L*. *boscai* presence (OR = 29.10, IC_95%_ [3.78–223.90]) (Table 2 and Fig 3). Distance to temporary streams did not affect strongly the occurrence of *L*. *boscai* (OR = 1.00, IC_95%_ [1.00–1.00]).

### Triturus marmoratus

*T*. *marmoratus* presence was significantly different between agriculture and *montados*, agriculture and native forest, eucalypt stands and *montados*, and eucalypt stands and native forests, at the intermediate (χ^2^ = 25.61, df = 3, P<0.001) and broad scales (χ^2^ = 23.51, df = 3, P<0.001) (Table 1; Table B in S1 File). Of the four top-ranked models, three included a combination of covariates at the different scales, and one included covariates exclusively from the local scale, accounting for 0.92 of the Akaike weights. There was little support for models at the intermediate and broad scale, with ΔAIC_c_>9.9 (ΔAIC_c_ Local scale < ΔAIC_c_ Broad scale ≈ ΔAIC_c_ Intermediate scale). The presence of predatory fish was the only variable whose confidence intervals did not overlap zero and had a negative effect on *T*. *marmoratus* occurrence (OR = 0.15, IC_95%_ [0.05–0.47]) (Table 2 and Fig 3).

### Pelobates cultripes

**Fig 4:**
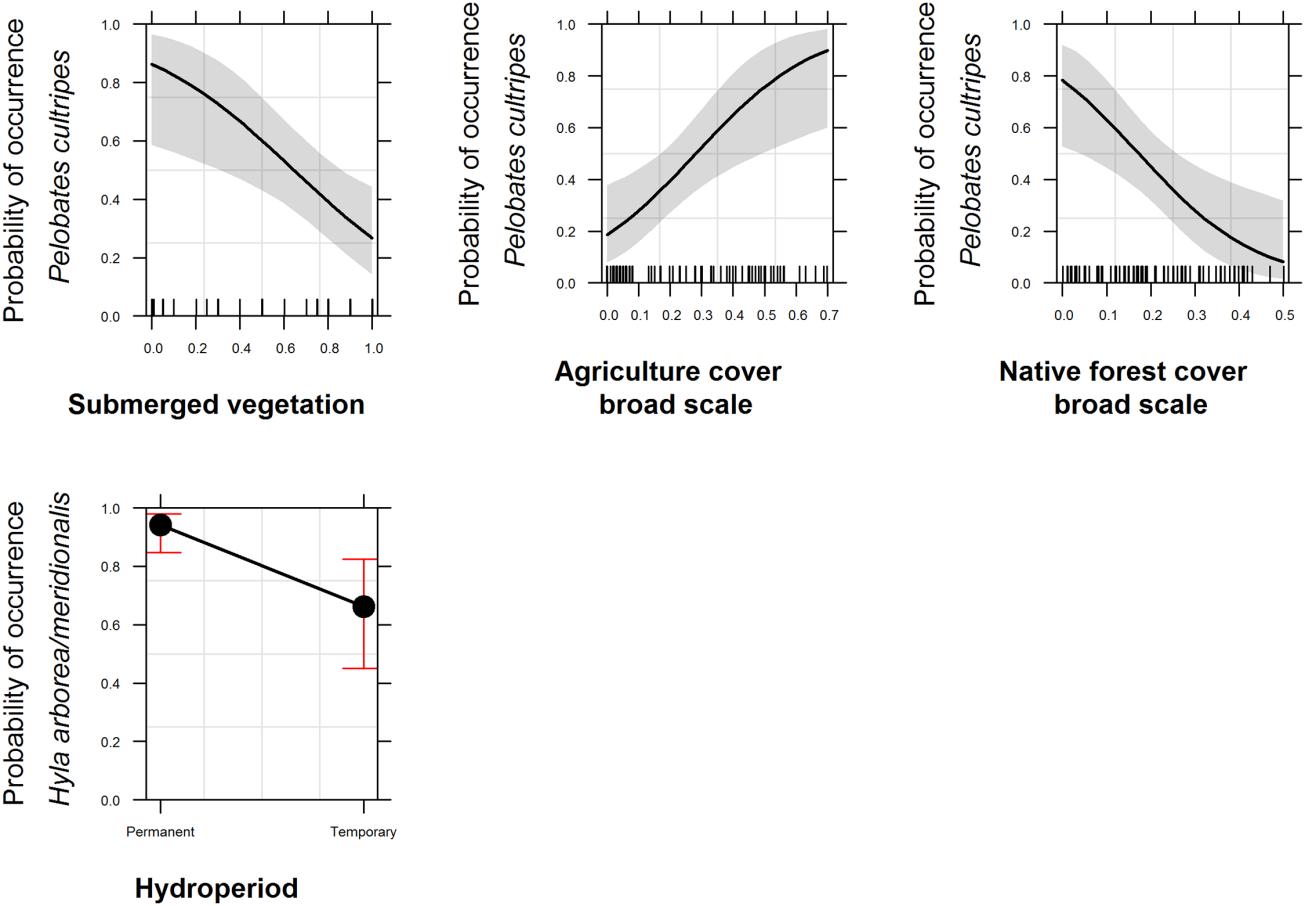
Fitted values predicted by the top ranked model for the anurans for each of the response variables with strong effect according to the odds ratio results. The dashed line are the confidence intervals at 95%.

*P*. *cultripes* presence was significantly different between agriculture and native forest, at both the intermediate (χ^2^ = 11.51, df = 3, P<0.05) and the broad scale (χ^2^ = 29.11, P<0.001) (Table 1; Table B in S1 File). At the broad scale, *P*. *cultripes* occurrence was also significantly different between agriculture and eucalypt stands, agriculture and *montados* and eucalypt and native forest (Table B in S1 File). A combination of local and broad-scale spatial covariates were included in the models that supported *P*. *cultripes* presence, namely the proportion of submerged aquatic vegetation, which had a negative effect on the presence of this anuran (OR = 0.06, IC_95%_ [7.85e^-03^-0.47]), and agricultural cover (positive effect) (OR = 182.12, IC_95%_ [3.25–1.02e^04^]) and native forest cover (negative effect) (OR = 0.001, IC_95%_ [3.94e^-06^-0.40]) at broad scale (Table 3 and Fig 4). The remaining scale models had a ΔAIC_c_>2 (ΔAIC_c_ Local scale ≈ ΔAIC_c_ Broad scale < ΔAIC_c_ Intermediate scale).

**Table 3:** Model averaged parameter estimates (β)(top-ranked models) for each of the anurans, odds ratio (OR) and respective 95% confidence intervals (IC_95%_).

	Pelobates cultripes			Hyla arborea/meridionalis		
	β	OR	IC_95%_	β	OR	IC_95%_
FISH				-1.21 (0.38)	0.30	0.04–1.98
FLOAT				1.24 (0.13)	3.45	0.58–20.59
SUBMER	**-2.80 (1)**	0.06	7.85e^-03^-0.47	**2.26 (0.78)**	9.57	1.09–83.90
HYDRO (temp)	-1.29 (1)	0.27	6.18–1.22e^-02^	**-2.341(1)**	0.10	0.02–0.53
SOIL	-0.58 (1)	0.56	0.10–3.04	0.56 (0.04)	1.75	0.29–10.64
NEPH				-0.02 (0.02)	0.98	0.97–1.00
NTEMP	-0.001 (0.37)	0.99	1.00–1.00			
NHY				-0.54 (0.02)	0.58	0.29–1.18
EUC400				-2.70 (0.03)	0.07	3.47e^-03^-1.30
AGRIC400				2.69 (0.17)	14.79	0.24–893.37
NATFOR400				-4.07 (0.03)	0.02	6.99e^-05^-4.19
MONT400				1.55 (0.02)	4.72	0.12–191.65
DTEMP400				-2.1e^-3^ (0.08)	0.99	0.99–1.00
AGRIC1000	**5.20 (0.91)**	182.12	3.25–1.02e^04^	4.15 (0.18)	63.71	0.41–9997.09
NATFOR1000	**-6.68 (0.91)**	0.001	3.94e^-06^-0.40			
EUC1000				**-2.35 (0.32)**	0.10	9.84e^-03^-0.93
DTEMP1000	3.2e^-5^ (0.19)	1.00	1.00–1.00			

Covariate importance between brackets. In bold are the covariates which confidence intervals do not overlap zero. Acronyms are explained in the text.

### Hyla arborea/meridionalis

*H*. *arborea/meridionalis* presence was significantly different between agriculture and native forest, and between eucalypt stands and native forest, at the intermediate (χ^2^ = 18.96, df = 3, P<0.001) and broad scales (χ^2^ = 34.02, df = 3, P<0.001). At the intermediate scale only, there were differences in presence between *montados* and native forest, and at the broad scale only, there were differences between agriculture and *montados* and eucalypt and *montados* (Table 1; Table B in S1 File).

A high number of models (21) accounted for 0.9 of Akaike weights, comprising models of combined scales and models with just local covariates (Table C in S1 File). There was a strong negative influence of temporary ponds (OR = 0.10, IC_95%_ [0.02–0.53]) as well as of eucalypt cover at the broad scale (OR = 0.10, IC_95%_ [0.001–0.93]) and a positive effect of submerged vegetation (OR = 9.57, IC_95%_ [1.09–83.90]) (Table 3 and Fig 4).

## Discussion

In summary, and considering the effect size of the variables, at the local scale, the presence of predatory fish had a consistently strong negative effect on the presence of Caudata. At this local scale, submerged vegetation had opposing influences on *L*. *boscai* and *H*. *arborea/meridionalis* (positive) and *P*. *cultripes* (negative) and *Hyla arborea/meridionalis* avoided temporary ponds. At the intermediate scale, distance to the nearest occupied pond by conspecifics had a strong negative effect on the presence of both *P*. *waltl* and *L*. *boscai*. Eucalypt cover had an intense positive effect on *S*. *salamandra* occurrence at both intermediate and broad scales, but had the opposite effect on *L*. *boscai* occurrence. *L*. *boscai* avoided also areas with high density of ponds, at the broad scale. At this scale also, the proportion of agriculture cover resulted in a strong positive influence on *P*. *waltl* and *P*. *cultripes* occurrences. Finally, still at the broad scale, *P*. *cultripes* strongly avoided native forests while *H*. *arborea/meridionalis* avoided eucalypt stands.

Some of our initial hypotheses were supported by our results. At a local scale, predatory fish presence was the single variable present in all top-ranked occurrence models for Caudata, having a negative influence on species occurrence. In addition, models with covariates across the three spatial scales had stronger support than models taken individually. However, some of our results were contrary to expectations. At the local scale, *H*. *arborea/meridionalis* preferred permanent ponds over temporary ponds and *P*. *cultripes* was not favoured by the increase of submerged vegetation. At the intermediate and broad scales, density of streams and/or ponds did not increase species occurrence and the decrease of distance of the connectivity variables did not show a consistent positive relationship with species occurrence. At the broad scale, eucalypt plantations had a negative effect on *L*. *boscai and H*. *arborea/meridionalis* occurrence, and had no negative effect on the other species, even favouring the presence of *S*. *salamandra*.

The negative impact of introduced predatory fish on amphibians has been reported in several studies worldwide [74–79] and in the Mediterranean region [36, 80–82]. These impacts occur through direct predation, competition or pathogen transfer [81, 83–87]. Certain amphibians may be more susceptible to these threats because they are not usually exposed to predatory fish, either because they are associated with temporary ponds, where fish are absent, and therefore lack appropriate defences [88] or because they do not recognise these fish as threats since they are all non-native species and they had little or no evolutionary history with these predators [87]. Nevertheless, there are amphibians that co-exist with alien fish, and have defence mechanisms either because they may have developed them during the course of evolution and conserve them in the absence of predators or because those mechanisms work against both native and non-native predators [87]. In this situation, tadpoles may show changes in morphological traits (e.g. increased tail area) and in behaviour (lower activity rate, aggregation, higher use of complex aquatic vegetation for refuge) to adapt to the novel situation besides tadpole unpalatability and/or chemically mediated predator avoidance [89–92]. Presence or absence of fish was irrelevant for the occurrence models for anurans, although other studies have detected a negative relationship (*P*. *cultripes*: [55]; *H*. *arborea*: [78, 84]). It has been reported in previous studies that these species are resilient against predatory fish due to their morphological traits or plasticity. Specifically, *P*. *cultripes* larvae attain a large body size (on average around 80 mm, [93]) and *H*. *arborea* larvae are able to develop deeper tail fins and deeper tail muscles in presence of fish [89]. On the other hand, the swimming behaviour of both these larvae, which are nektonic, may increase the chance of being preyed upon by making them more visible to visually oriented predatory fish like *L*. *gibbosus* [84, 94–96].

Avoidance of temporary ponds by *H*. *arborea/meridionalis* has also been reported previously, e.g. [97]. Both species have a long larval stage, on average 3 months [98], and temporary ponds can dry out before metamorphosis is complete [99]. Although temporary ponds cannot support predatory fish, the desiccation risk in the Mediterranean region is high, so the preference for ponds with a long and stable hydroperiod may still improve the recruitment success of these amphibian species [39].

Aquatic vegetation can provide refuge, food [94] and protection against UV-B radiation, which can affect some species during early developmental stages [100]. Three of the studied species occurrences were affected differently by aquatic vegetation. *L*. *boscai* and *H*. *arborea/meridionalis* were found more in ponds with a high percentage of vegetation, which is likely to be related to the oviposition behaviour shown by newts of wrapping each egg individually in leaves to protect them from UV-B radiation and predators [40]. In contrast, the anuran *P*. *cultripes* avoided ponds with a high percentage of submerged vegetation. *P*. *cultripes* is a good swimmer, feeds within the water column, and it is possible that too much aquatic vegetation interferes with its foraging.

Connectivity covariates—distance to streams or ponds and density of streams and ponds—showed different trends amongst the studied species, and only *P*. *cultripes* and *T*. *marmoratus* occurrence were not affected by these covariates. The increased probability of occurrence of a certain species in a pond was often related to a decrease in the distance to the nearest other pond occupied by that same species (*P*. *waltl* and *L*. *boscai*). Most juvenile Caudata do not disperse more than 500 metres from the breeding pond and adults show a high level of site fidelity to the pond they first reproduced in [31]. In fact, juveniles are not well adapted to dispersal. They are smaller, more prone to desiccation and have less locomotor capacity than adults to travel long distances, and they sustain high mortality rates when they leave the pond [25]. Due to all these constraints it is most likely that juvenile Caudata disperse to nearby ponds, depending on close “networks” of ponds where the species is already present [101]. Chemical cues, both aquatic and terrestrial, may lead these juveniles to non-natal ponds [30, 31]. In a laboratory setting, *L*. *boscai* preferred water that contained chemical cues of themselves or conspecifics [30]. Heterospecific auditory cues may also attract some species and help with pond orientation. *T*. *marmoratus* showed positive phonotactic orientation when exposed to *Bufo calamita* advertisement calls [102], and *Lissotriton helveticus* showed the same behaviour when exposed to *P*. *perezi* calls [103]. *Triturus alpestris* is capable of long distance homing using only magnetic compass [104]. However, a high density of ponds had a negative impact on *L*. *boscai* occurrence. Our results are partially coincident with those by [105], with species being more abundant at intermediate pond density. Both local (aquatic; within-pond) and landscape (terrestrial) features are expected to influence species occurrence. The contribution of each feature may depend on their spatial configuration and quality [106]. Water is a scarce resource in Mediterranean regions, so aquatic habitats are expected to act as a constraint to population occurrence and dynamics, with the distribution and characteristics of terrestrial habitats only having a major role when ponds are plentiful [105].

*S*. *salamandra* is often associated with temporary streams for breeding, avoiding ephemeral streams [107], contrary to our results. This forest species has a long terrestrial phase, using ponds or streams only to deposit their larvae, spending the rest of their time on land. Our results showed also the positive effect of eucalypt plantation at both intermediate and broad scale on the occurrence of this species. Eucalypt plantations place large demands on soil water. In some cases water depletion caused by eucalypts can reach 8 metres depth [19], leading to low levels of moisture at the surface. Eucalypt plantations in the Mediterranean region are also characterised by a lower macro-arthropod abundance when compared to native habitats such as cork oak woodlands, olive groves or riparian vegetation [108]. Despite this, eucalypt plantations apparently favoured *S*. *salamandra* occurrence, a species that has a strong association with high woodland cover [109], and these stands are the only forest-cover type that cover extensive areas in the region. We tentatively interpret this to be a consequence of the proximity of ephemeral streams which may supply sufficient humidity to reduce the risk of desiccation and also serve as a source of prey, especially if the original riparian vegetation is maintained [108], which was verified in most of our study area.

Nevertheless, the conditions that favoured *S*. *salamandra* had the opposite effect on *L*. *boscai*. This species, although being one of the most aquatic European newts, makes terrestrial incursions throughout the year and goes into summer dormancy in hot and dry regions [110]. *L*. *boscai* has a low ecological plasticity [111] and low dispersal ability [40]. Thus, any additional barrier, like chemical fertilisers, soil disturbances and low soil depth can add costs to the distance travelled [33, 112]. In addition to the impact of eucalypt plantations in causing soil water depletion, the smaller size of this newt compared with *S*. *salamandra*, may make it more susceptible to predation and desiccation when crossing extensive areas of exotic stands.

Agricultural land is often associated negatively with amphibian presence due to multiple interventions throughout the year, altering the soil humidity and jeopardizing refuges during aestivation, as well as potentially causing direct mortality due to injuries [113]. The use of fertilisers may also affect the body condition of amphibians and their ability to disperse depending upon concentrations, time of the year and species sensitivity [114, 115]. In addition, cattle grazing may have a negative impact on water quality through nitrogenous deposits, increasing eutrophication, degrading water quality but also by grazing on the shoreline vegetation, that acts as refuge and source of food and oviposition sites [116, 117]. However, the agricultural use in the study area is extensive, and comprises olive groves, wheat areas, and small-scale farming for personal use, with low use of fertilisers and grazing at low densities, mainly by cattle. Hence, the practice of small-scale agriculture, which represents an anthropogenic disturbance of only intermediate impact, favoured the occurrence of *P*. *waltl* and *P*. *cultripes*, especially at the broad scale. Nonetheless, that was not the case for native forests, which combined pine, oak and mixed forests, and *P*. *cultripes* avoided these areas. Adult *P*. *cultripes* need soft soils to dig their burrows and they might have some difficulties digging in forested areas when compared with agricultural land.

Overall, our results showed that eucalypt stands had a negative impact on the occurrence of *L*. *boscai* and *H*. *arborea/meridionalis*, whilst having a positive effect on *S*. *salamandra* occurrence. For the remaining species, eucalypt cover was an unimportant factor compared with other landscape and local variables, especially the presence of predatory fish, which had a strong negative impact on the occurrence of Caudata The eucalypt stands of the study area were embedded in a traditional agro-forestry matrix, with intermediate disturbance, and a conservative approach must be taken to extrapolate these results to larger extensions of eucalypt plantations surrounded by a degraded matrix, with a high level of disturbance (e.g. intensive agriculture, barriers such as roads). Therefore, sensitive management of these plantations in terms of conservation is advised. As further research, we suggest the evaluation of functional connectivity. This was not possible in our study because there are only a few studies that relate costs of travelling with habitat structure, and to our knowledge, for some species, there is an absolute lack of information, like for *P*. *cultripes* or *L*. *boscai*. In conclusion, eucalypts had limited effects on the amphibian community at the migration and dispersal scales, but fish presence had a major impact at all scales. Our results highlight the importance of context-dependency in predicting impacts of landscape composition and structure on amphibian populations. However, the over-riding importance of fish as a negative impact suggests that forest managers should prevent new introductions fish and other exotic predators and eradicate fish from already-occupied ponds whenever possible. When fish eradication is not possible, creation of new permanent fish-free ponds nearby fish-occupied ponds may be an alternative strategy.

## Supporting Information

S1 File Full models built for the six species studied. For the local, intermediate and broad scales the models were the same for each of the six species. For the combined scale they differ as explained by the procedure in Fig 2 (Table A). Chi-square results, between pairs of land cover and species occurrence at the intermediate and broad scale Significant difference obtained if p<0.05 (in bold) (Table B). Model selection results for analysis of the species occurrence. Models which AICc weight sums up to 0.9 or more are shown, as well as the two highest ranked models at each spatial scale and the reference if they were used in the model average (✓). For each response variable is presented the model description, the number of estimable parameters (K), the sample-size adjusted AIC (AIC_*c*_), Akaike differences (ΔAIC_*c*_), Akaike weights and the log-likelihood (logLik). In bold are the covariates which confidence intervals do not overlap zero. All models have the covariate subset added as a random variable (Table C).
(DOCX)
