# Supplementary material for: Exotic Fish in Exotic Plantations: A Multi-Scale Approach to Understand Amphibian Occurrence in the Mediterranean Region
Source: PLoS One. 2015 Jun 10;10(6):e0129891. doi: 10.1371/journal.pone.0129891 (PMC4465696; doi:10.1371/journal.pone.0129891)
Supplement: S1 File — For the local, intermediate and broad scales the models were the same for each of the six species. For the combined scale they differ as explained by the procedure in Fig 2 (Table A). Chi-square results, between pairs of land cover and species occurrence at the intermediate and broad scale Significant difference obtained if p<0.05 (in bold) (Table B). Model selection results for analysis of the species occurrence. Models which AICc weight sums up to 0.9 or more are shown, as well as the two highest ranked models at each spatial scale and the reference if they were used in the model average (✓). For each response variable is presented the model description, the number of estimable parameters (K), the sample-size adjusted AIC (AICc), Akaike differences (ΔAICc), Akaike weights and the log-likelihood (logLik). In bold are the covariates which confidence intervals do not overlap zero. All models have the covariate subset added as a random variable (Table C). (DOCX) [file pone.0129891.s001.docx]

**Supporting Information**

**Table A. Full models built for the six species studied.**  For the local, intermediate and broad scales the models were the same for each of the six species. For the combined scale they differ as explained by the procedure in Fig.2

| Response variable | Scale |  |
| --- | --- | --- |
| All studied amphibians | Local | FISH+HYDRO+SOIL+FLOAT+EMER+SUBMER |
|  | Intermediate | DPOND400+DEPH400+DTEMP400+NATFOR400+EUC400 +NPW* |
|  |  | DPOND400+DEPH400+DTEMP400+NATFOR400+MONT400+AGRIC400 +NPW* |
|  |  | DPOND400+DEPH400+DTEMP400 +NPW* |
|  |  | NATFOR400+MONT400+AGRIC400 +NPW* |
|  |  | EUC400 +NPW* |
|  |  | AGRIC400 +NPW* |
|  |  | MONT400+NPW* |
|  |  | EUC400+NPW* |
|  |  | AGRIC400+NPW* |
|  |  | MONT400+NPW |
|  | Broad | DEPH1000+DTEMP1000+DPOND1000+NATFOR1000+EUC1000+NPW* |
|  |  | DEPH1000+DTEMP1000+DPOND1000+NATFOR1000+MONT1000+AGRIC1000+NPW* |
|  |  | DEPH1000+DTEMP1000+DPOND1000+NPW* |
|  |  | NATFOR1000+MONT1000+AGRIC1000+NPW* |
|  |  | EUC1000+NPW* |
|  |  | AGRIC1000+NPW* |
|  |  | MONT1000+NPW* |
|  |  | EUC1000+NPW* |
|  |  | AGRIC1000+NPW* |
|  |  | MONT1000+NPW* |
|  | Combined |  |
| *Pleurodeles waltl* |  | AGRIC1000+NPW+NEPH+NPOND+NTEMP+NEPH:NPOND+FISH |
|  |  | AGRIC1000+NPW+NEPH+NPOND+NEPH:NPOND+FISH |
|  |  | AGRIC1000+NPW+NATFOR1000+NEPH+NPOND+NTEMP+NEPH:NPOND+FISH |
|  |  | AGRIC1000+NPW+MONT1000+NEPH+NPOND+NTEMP+NEPH:NPOND+FISH |
|  |  | AGRIC1000+NPW+NEPH+NPOND+NTEMP+NEPH:NPOND |
|  |  | AGRIC1000+NPW+NEPH+NPOND+NEPH:NPOND |
|  |  | AGRIC1000+NPW+NATFOR1000+NEPH+NPOND+NTEMP+NEPH:NPOND |
|  |  | AGRIC1000+NPW+MONT1000+NEPH+NPOND+NTEMP+NEPH:NPOND |
| *Salamandra salamandra* |  | EUC400+NEPH+FISH |
|  |  | EUC1000+NEPH+FISH |
|  |  | EUC400+NEPH+NPOND+FISH |
|  |  | EUC1000+NEPH+NTEMP+FISH |
|  |  | EUC400+NEPH+NTEMP+FISH |
|  |  | EUC1000+NATFOR1000+NEPH+FISH |
|  |  | EUC1000+NATFOR1000+NEPH+NTEMP+FISH |
|  |  | EUC400+NATFOR400+NEPH+FISH |
|  |  | EUC400+NEPH+NPOND+NTEMP+FISH |
|  |  | EUC400+NEPH |
|  |  | EUC1000+NEPH |
|  |  | EUC400+NEPH+NPOND |
|  |  | EUC1000+NEPH+NTEMP |
|  |  | EUC400+NEPH+NTEMP |
|  |  | EUC1000+NATFOR1000+NEPH |
|  |  | EUC1000+NATFOR1000+NEPH+NTEMP |
|  |  | EUC400+NATFOR400+NEPH |
|  |  | EUC400+NEPH+NPOND+NTEMP |
| *Lissotriton boscai* |  | AGRIC400+NLB+MONT400+AGRIC400:MONT400+FISH |
|  |  | AGRIC400+NLB+MONT400+NTEMP+AGRIC400:MONT400+FISH |
|  |  | AGRIC400+NLB+MONT400+DEPH400+AGRIC400:MONT400+FISH |
|  |  | NLB+FISH |
|  |  | NLB+NATFOR400+FISH |
|  |  | AGRIC400+NLB+MONT400+DPOND400+AGRIC400:MONT400+FISH |
|  |  | NLB+NTEMP+FISH |
|  |  | AGRIC400+NLB+MONT400+NATFOR400+AGRIC400:MONT400+FISH |
|  |  | NLB+DTEMP1000+FISH |
|  |  | NLB+DPOND1000+DTEMP1000+FISH |
|  |  | AGRIC1000+NLB+DPOND1000+DTEMP1000+FISH |
|  |  | EUC1000+NLB+DPOND1000+DTEMP1000+FISH |
|  |  | AGRIC1000+NLB+NATFOR1000+DPOND1000+DTEMP1000+AGRIC1000:NATFOR1000+FISH |
|  |  | AGRIC400+NLB+MONT400+AGRIC400:MONT400+FISH+SUBMER |
|  |  | AGRIC400+NLB+MONT400+NTEMP+AGRIC400:MONT400+FISH+SUBMER |
|  |  | AGRIC400+NLB+MONT400+DEPH400+AGRIC400:MONT400+FISH+SUBMER |
|  |  | NLB+FISH+SUBMER |
|  |  | NLB+NATFOR400+FISH+SUBMER |
|  |  | AGRIC400+NLB+MONT400+DPOND400+AGRIC400:MONT400+FISH+SUBMER |
|  |  | NLB+NTEMP+FISH+SUBMER |
|  |  | AGRIC400+NLB+MONT400+NATFOR400+AGRIC400:MONT400+FISH+SUBMER |
|  |  | NLB+DTEMP1000+FISH+SUBMER |
|  |  | NLB+DPOND1000+DTEMP1000+FISH+SUBMER |
|  |  | AGRIC1000+NLB+DPOND1000+DTEMP1000+FISH+SUBMER |
|  |  | EUC1000+NLB+DPOND1000+DTEMP1000+FISH+SUBMER |
|  |  | AGRIC1000+NLB+NATFOR1000+DPOND1000+DTEMP1000+AGRIC1000:NATFOR1000+FISH+SUBMER |
| *Triturus marmoratus* |  | FISH+FLOAT+NTM |
|  |  | FISH+FLOAT+SUBMER+NTM |
|  |  | EMER+FISH+FLOAT+NTM |
| *Pelobates cultripes* |  | HYDRO+SOIL+SUBMER+AGRIC400 |
|  |  | HYDRO+SOIL+SUBMER+AGRIC1000+NATFOR1000 |
|  |  | HYDRO+SOIL+SUBMER+AGRIC1000+NATFOR1000 |
|  |  | FLOAT+HYDRO+SOIL+SUBMER+AGRIC400 |
|  |  | FLOAT+HYDRO+SOIL+SUBMER+AGRIC1000+NATFOR1000 |
|  |  | FLOAT+HYDRO+SOIL+SUBMER+AGRIC1000+NATFOR1000 |
|  |  | AGRIC400+MONT400+HYDRO+SOIL+SUBMER |
|  |  | EUC400+NATFOR400+HYDRO+SOIL+SUBMER |
|  |  | AGRIC400+HYDRO+SOIL+SUBMER |
|  |  | AGRIC400+MONT400+DPOND400+HYDRO+SOIL+SUBMER |
|  |  | AGRIC400+DPOND400+HYDRO+SOIL+SUBMER |
|  |  | AGRIC400+MONT400+NPOND+HYDRO+SOIL+SUBMER |
|  |  | AGRIC400+NATFOR400+HYDRO+SOIL+SUBMER |
|  |  | EUC400+NATFOR400+NPOND+HYDRO+SOIL+SUBMER |
|  |  | EUC400+HYDRO+SOIL+SUBMER |
|  |  | AGRIC400+MONT400+NATFOR400+HYDRO+SOIL+SUBMER |
|  |  | EUC400+NATFOR400+DPOND400+HYDRO+SOIL+SUBMER |
|  |  | AGRIC1000+NATFOR1000+HYDRO+SOIL+SUBMER |
|  |  | AGRIC1000+NATFOR1000+NPOND+HYDRO+SOIL+SUBMER |
|  |  | AGRIC1000+HYDRO+SOIL+SUBMER |
|  |  | AGRIC1000+NATFOR1000+DPOND1000+HYDRO+SOIL+SUBMER |
|  |  | AGRIC1000+NATFOR1000+DTEMP1000+HYDRO+SOIL+SUBMER |
|  |  | AGRIC1000+NATFOR1000+NTEMP+HYDRO+SOIL+SUBMER |
|  |  | AGRIC1000+MONT1000+NATFOR1000+HYDRO+SOIL+SUBMER |
|  |  | AGRIC1000+MONT1000+NATFOR1000+NPOND+HYDRO+SOIL+SUBMER |
| *Hyla arborea/meridionalis* |  | HYDRO+SUBMER+AGRIC400 |
|  |  | HYDRO+SUBMER+AGRIC1000 |
|  |  | HYDRO+SUBMER+AGRIC400 +EUC1000 |
|  |  | FISH+HYDRO+SUBMER+AGRIC400 |
|  |  | FISH+HYDRO+SUBMER+AGRIC1000 |
|  |  | FISH+HYDRO+SUBMER +EUC1000 |
|  |  | AGRIC400+NHY+HYDRO |
|  |  | EUC400+NATFOR400+NEPH+HYDRO |
|  |  | AGRIC400+MONT400+NEPH+HYDRO |
|  |  | AGRIC400+MONT400+NEPH+HYDRO |
|  |  | AGRIC400+NHY+MONT400+NEPH+HYDRO |
|  |  | AGRIC400+DTEMP400+HYDRO |
|  |  | EUC400+NHY+NATFOR400+NEPH+HYDRO |
|  |  | AGRIC400+MONT400+DTEMP400+HYDRO |
|  |  | EUC400+NATFOR400+HYDRO |
|  |  | AGRIC400+NHY+NEPH+NPOND+HYDRO |
|  |  | AGRIC400+NHY+DPOND400+HYDRO |
|  |  | AGRIC400+NHY+NEPH+NTEMP+HYDRO |
|  |  | AGRIC400+MONT400+NEPH+NTEMP+HYDRO |
|  |  | AGRIC400+NHY+NPOND+HYDRO |
|  |  | AGRIC400+HYDRO |
|  |  | AGRIC400+MONT400+HYDRO |
|  |  | AGRIC400+NHY+MONT400+HYDRO |
|  |  | EUC400+NATFOR400+NEPH+NTEMP+HYDRO |
|  |  | AGRIC400+NHY+NTEMP+HYDRO |
|  |  | EUC400+NATFOR400+DTEMP400+HYDRO |
|  |  | EUC400+NEPH+HYDRO |
|  |  | EUC400+NHY+NEPH+HYDRO |
|  |  | EUC400+NHY+NATFOR400+HYDRO |
|  |  | EUC400+NHY+HYDRO |
|  |  | AGRIC400+NHY+DPOND400+DTEMP400+HYDRO |
|  |  | EUC400+HYDRO |

*NPW is used as an example, using distance to nearest pond where conspecific is present. For the remaining five species, we replaced it with the respective data for the studied species.

**Table B. Chi-square results, between pairs of land cover and species occurrence at the intermediate and broad scale** Significant difference obtained if p<0.05 (in bold)

|  |  | AGRIC | EUC | MONT | NATFOR | AGRIC | EUC | MONT | NATFOR |
| --- | --- | --- | --- | --- | --- | --- | --- | --- | --- |
|  |  | Intermediate | | | | Broad | | | |
| *Pleurodeles waltl* | AGRIC | - | - | - | - | - | - | - | - |
|  | EUC | 0.72 | - | - | - | 0.17 | - | - | - |
|  | MONT | 0.08 | 0.22 | - | - | **0.0001** | **0.02** | - | - |
|  | NATFOR | **0.01** | **0.03** | 0.56 | - | **0.00** | **0.0004** | 0.33 | - |
| *Salamandra salamandra* | AGRIC | - | - | - | - | - | - | - | - |
|  | EUC | **0.001** | - | - | - | **0.0013** | - | - | - |
|  | MONT | 0.10 | **0.00** | - | - | 0.06 | **0.00** | - | - |
|  | NATFOR | 0.21 | **0.00** | 1.00 | - | **0.02** | **0.00** | 1.00 | - |
| *Lissotriton boscai* | AGRIC | - |  |  |  | - | - | - | - |
|  | EUC | **0.03** | - |  |  | 0.06 | - | - | - |
|  | MONT | 0.84 | **0.01** | - |  | 0.05 | **0.0001** | - | - |
|  | NATFOR | **0.01** | **0.00** | **0.04** | - | **0.002** | **0.00** | 0.37 | - |
| *Triturus marmoratus* | AGRIC | - |  |  |  | - | - | - | - |
|  | EUC | 0.47 | - |  |  | 1.00 | - | - | - |
|  | MONT | **0.0004** | **0.00** | - |  | **0.005** | **0.008** | - | - |
|  | NATFOR | **0.02** | **0.002** | 0.28 | - | **0.0001** | **0.0002** | 0.37 | - |
| *Pelobates cultripes* | AGRIC | - |  |  |  | - | - | - | - |
|  | EUC | 0.09 | - |  |  | **0.02** | - | - | - |
|  | MONT | 0.09 | 1.00 | - |  | **0.0003** | 0.28 | - | - |
|  | NATFOR | **0.002** | 0.25 | 0.25 | **-** | **0.00** | **0.03** | 0.44 | - |
| *Hyla arborea/meridionalis* | AGRIC | - |  |  |  | - | - | - | - |
|  | EUC | 0.87 | - |  |  | 1.00 | - | - | - |
|  | MONT | 0.15 | 0.08 | - |  | **0.0005** | **0.0009** | - | - |
|  | NATFOR | **0.0003** | **0.0001** | **0.04** | - | **0.00** | **0.00** | 0.28 | - |

AGRIC – agricultural; EUC – eucalypt plantations; MONT – *montados*; NATFOR – native forests

**Table C. Model selection results for analysis of the species occurrence**. Models which AICc weight sums up to 0.9 or more are shown, as well as the two highest ranked models at each spatial scale and the reference if they were used in the model average (✓). For each response variable is presented the model description, the number of estimable parameters (K), the sample-size adjusted AIC (AIC*_c_*), Akaike differences (∆AIC*_c_*), Akaike weights and the log-likelihood (logLik). In bold are the covariates which confidence intervals do not overlap zero. All models have the covariate subset added as a random variable.

| Response variable | Models used in model averaged | Scale | Model | k | AIC_c_ | ∆AIC_c_ | Akaike weight | logLik |
| --- | --- | --- | --- | --- | --- | --- | --- | --- |
| *Pleurodeles waltl* |  | | | | | | | |
|  | ✓ | Combined | **FISH+NPW+NEPH+NPOND+NEPH:NPOND+AGRIC1000** | 8 | 79.7 | 0.00 | 0.60 | -30.96 |
|  | ✓ | Combined | **FISH+NPW+NEPH+NPOND**+NTEMP**+NEPH:NPOND+AGRIC1000** | 9 | 80.8 | 1.07 | 0.35 | -30.25 |
|  |  | Broad | **NPW+NEPH+NPOND**+NTEMP**+NEPH:NPOND+AGRIC1000** | 8 | 86.6 | 6.88 | 0.02 | -34.39 |
|  |  | Broad | **NPW+NEPH+NPOND+NEPH:NPOND+AGRIC1000** | 7 | 87.1 | 7.38 | 0.02 | -35.86 |
|  |  | Local | **FISH**+SOIL**+EMER**+FLOAT | 6 | 89.5 | 9.80 | 0.004 | -38.25 |
|  |  | Local | **FISH**+EMER | 4 | 89.8 | 10.10 | 0.004 | -40.68 |
|  |  | Intermediate | **NPW**+DEPH400+AGRIC400 | 5 | 96.9 | 17.15 | 0.000 | -43.07 |
|  |  | Intermediate | **NPW**+DTEMP400 | 4 | 97.7 | 17.92 | 0.000 | -44.59 |
| *Salamandra salamandra* |  | | | | | | | |
|  | ✓ | Combined | **FISH+NEPH+EUC1000** | 5 | 92.1 | 0.00 | 0.37 | -40.67 |
|  | ✓ | Combined | **FISH+NEPH+EUC400** | 5 | 92.5 | 0.43 | 0.30 | -40.88 |
|  | ✓ | Combined | **FISH+NEPH+EUC1000**+NATFOR1000 | 6 | 92.7 | 0.58 | 0.27 | -39.81 |
|  |  | Intermediate | **NEPH+EUC400** | 4 | 97.7 | 5.58 | 0.02 | -44.59 |
|  |  | Broad | **NEPH+EUC1000** | 4 | 98.2 | 6.18 | 0.02 | -44.88 |
|  |  | Intermediate | **NEPH**+NPOND**+EUC400** | 5 | 98.7 | 6.64 | 0.01 | -43.99 |
|  |  | Broad | **NEPH**+NTEMP**+EUC1000** | 5 | 98.8 | 6.71 | 0.01 | -44.02 |
|  |  | Local | **FISH+SOIL**+SUBMER | 5 | 107.8 | 15.75 | 0.00 | -48.54 |
|  |  | Local | **FISH+SOIL**+HYDRO | 5 | 108.3 | 16.18 | 0.00 | -48.76 |
| *Lissotriton boscai* |  | | | | | | | |
|  |  | Combined | **FISH+SUBMER+NLB+DPOND1000+DTEMP1000+EUC1000** | 8 | 89.1 | 0.00 | 0.99 | -35.66 |
|  |  | Broad | **NLB+DTEMP1000** | 4 | 99.9 | 10.80 | <0.01 | -45.73 |
|  |  | Broad | **NLB**+DPOND1000**+DTEMP1000** | 5 | 100.2 | 11.05 | <0.01 | -44.73 |
|  |  | Intermediate | **NLB+AGRIC400**+MONT400**+AGRIC400:MONT400** | 6 | 102.2 | 13.05 | <0.01 | -44.58 |
|  |  | Intermediate | **NLB**+NTEMP**+AGRIC400**+MONT400**+AGRIC400:MONT400** | 7 | 102.4 | 12.28 | <0.01 | -43.51 |
|  |  | Local | **FISH+SUBMER** | 4 | 106.1 | 16.99 | 0.000 | -48.82 |
|  |  | Local | **FISH**+SOIL**+SUBMER** | 5 | 106.5 | 17.40 | 0.000 | -47.90 |
| *Triturus marmoratus* |  | | | | | | | |
|  | ✓ | Combined | **FISH**+FLOAT**+NTM** | 5 | 100.9 | 0.00 | 0.38 | -45.08 |
|  | ✓ | Combined | **FISH**+FLOAT+EMER+NTM | 6 | 101.9 | 1.05 | 0.22 | -44.45 |
|  | ✓ | Combined | **FISH**+FLOAT+SUBMER+NTM | 6 | 102.4 | 1.53 | 0.18 | -44.69 |
|  | ✓ | Local | **FISH+FLOAT** | 4 | 102.8 | 1.94 | 0.14 | -47.17 |
|  |  | Local | **FISH**+FLOAT+SUBMER | 5 | 104.1 | 3.23 | 0.08 | -46.69 |
|  |  | Broad | **NTM**+AGRIC1000 | 4 | 110.8 | 9.92 | <0.01 | -51.16 |
|  |  | Broad | **NTM** | 3 | 111.4 | 10.49 | <0.01 | -52.54 |
|  |  | Intermediate | **NTM**+DPOND400 | 4 | 112.2 | 11.36 | <0.01 | -51.88 |
|  |  | Intermediate | **NTM**+AGRIC400 | 4 | 112.5 | 11.63 | <0.01 | -52.02 |
| *Pelobates cultripes* |  | | | | | | | |
|  | ✓ | Combined | **SUBMER**+SOIL+HYDRO+NTEMP**+AGRIC1000+NATFOR1000** | 8 | 97.0 | 0.00 | 0.34 | -39.57 |
|  | ✓ | Combined | **SUBMER**+SOIL+HYDRO**+AGRIC1000**+NATFOR1000 | 7 | 97.1 | 0.12 | 0.32 | -40.84 |
|  | ✓ | Combined | **SUBMER**+SOIL+HYDRO+DTEMP1000**+AGRIC1000+NATFOR1000** | 8 | 98.3 | 1.29 | 0.18 | -40.22 |
|  | ✓ | Combined | **SUBMER**+SOIL+HYDRO+DPOND+**AGRIC1000**+NATFOR1000 | 5 | 99.8 | 2.82 | 0.08 | -44.53 |
|  |  | Broad | NPOND**+AGRIC1000+NATFOR1000** | 5 | 101.7 | 4.69 | 0.03 | -45.46 |
|  |  | Local | **SUBMER**+FLOAT**+SOIL+HYDRO** | 6 | 101.8 | 4.79 | 0.03 | -44.36 |
|  |  | Broad | **AGRIC1000** | 3 | 102.0 | 5.05 | 0.03 | -47.87 |
|  |  | Intermediate | **AGRIC400**+MONT400 | 4 | 107.1 | 10.10 | <0.01 | -49.29 |
|  |  | Intermediate | **EUC400**+NATFOR400 | 4 | 107.5 | 10.55 | <0.01 | -49.52 |
| *Hyla arborea/meridionalis* |  |  | | |  |  |  |  |
|  | ✓ | Combined | **SUBMER+HYDRO+EUC1000** | 5 | 65.8 | 0.00 | 0.19 | -27.55 |
|  | ✓ | Combined | **SUBMER+HYDRO+**FISH**+EUC1000** | 6 | 66.7 | 0.84 | 0.13 | -26.81 |
|  | ✓ | Combined | **SUBMER+HYDRO+**AGRIC1000 | 5 | 66.9 | 1.10 | 0.11 | -28.10 |
|  | ✓ | Combined | FISH**+SUBMERGED+HYDROPERIOD**+AGRIC1000 | 6 | 67.8 | 2.03 | 0.07 | -27.41 |
|  | ✓ | Local | **SUBMER+HYDRO** | 4 | 68.4 | 2.56 | 0.05 | -29.95 |
|  | ✓ | Local | **SUBMER+HYDRO+**FISH | 5 | 68.5 | 2.71 | 0.05 | -28.90 |
|  | ✓ | Combined | **SUBMER+HYDRO** +AGRIC400 | 5 | 68.9 | 3.12 | 0.04 | -29.10 |
|  | ✓ | Combined | **HYDRO+**AGRIC400+**DTEMP400** | 5 | 68.9 | 3.17 | 0.04 | -29.13 |
|  | ✓ | Local | SUBMER**+HYDRO**+FLOAT | 5 | 69.2 | 3.41 | 0.04 | -29.25 |
|  | ✓ | Local | **HYDRO**+FLOAT | 4 | 69.2 | 3.43 | 0.03 | -30.38 |
|  | ✓ | Combined | **SUBMER+HYDRO**+FISH+AGRIC400 | 6 | 69.4 | 3.60 | 0.03 | -28.19 |
|  | ✓ | Local | FISH+FLOAT**+HYDRO** | 5 | 69.5 | 3.72 | 0.03 | -29.40 |
|  | ✓ | Local | FISH+SUBMER+FLOAT**+HYDRO** | 6 | 69.7 | 3.85 | 0.03 | -28.32 |
|  | ✓ | Combined | **HYDRO**+AGRIC400+NHY | 5 | 70.1 | 4.25 | 0.02 | -29.67 |
|  | ✓ | Local | FISH+**SUBMER**+SOIL**+HYDRO** | 6 | 70.3 | 4.44 | 0.02 | -28.61 |
|  | ✓ | Local | FISH**+HYDRO** | 4 | 70.4 | 4.55 | 0.02 | -30.94 |
|  | ✓ | Local | **SUBMER**+SOIL**+HYDRO** | 5 | 70.4 | 4.55 | 0.02 | -29.82 |
|  | ✓ | Combined | **HYDRO+**DTEMP400+EUC400+NATFOR400 | 6 | 70.5 | 4.66 | 0.02 | -28.72 |
|  | ✓ | Combined | **HYDRO**+AGRIC400+DTEM400+MONT400 | 6 | 70.5 | 4.71 | 0.02 | -28.75 |
|  | ✓ | Combined | **HYDRO+EUC400**+NATFOR400+NEPH | 6 | 70.9 | 5.02 | 0.02 | -28.90 |
|  | ✓ | Combined | **HYDRO**+AGRIC400 | 4 | 71.0 | 5.20 | 0.01 | -31.27 |
|  |  | Broad | NHY+NEPH**+AGRIC1000** | 5 | 73.2 | 7.38 | <0.01 | -31.22 |
|  |  | Broad | NHY**+AGRIC1000** | 4 | 73.3 | 7.48 | <0.01 | -32.39 |
|  |  | Intermediate | NHY**+AGRIC400** | 4 | 75.9 | 10.08 | <0.01 | -33.70 |
|  |  | Intermediate | NEPH**+EUC400**+NATFOR400 | 5 | 75.9 | 10.08 | <0.01 | -32.58 |

FISH - presence of predator fish (FISH); HYDRO – hydroperiod; SOIL - soil type (muddy or shale); FLOAT - % of floating aquatic vegetation; EMER - % of emergent aquatic vegetation; SUBMER - % of submersed aquatic vegetation; Proportion of land cover(AGRIC –agriculture; EUC – eucalypt plantations; MONT – montados; NATFOR – native forest); NEPH - the distance to the nearest ephemeral stream (m); NTEMP - the distance to the nearest temporary stream (m); NPOND – distance to ponds (m); DEPH – density of ephemeral streams (intermediate scale number per 50 ha; broad scale: number per 314 ha); DTEMP - density of temporary streams (intermediate scale number per 50 ha; broad scale: number per 314 ha); DPOND - density of ponds (intermediate scale number per 50 ha; broad scale: number per 314 ha); NPW – distance to nearest occupied pond with *Pleurodeles waltl* (m); NSS - distance to nearest occupied pond with *Salamandra salamandra* (m); NLB– distance to nearest occupied pond with *Lissotriton boscai* (m); NTM – distance to nearest occupied pond with *Triturus marmoratus* (m); NPC – distance to nearest occupied pond with *Pelobates cultripes* (m); NHY – distance to nearest occupied pond with *Hyla* spp. (m)).
